# Meditation Experience Predicts Introspective Accuracy

**DOI:** 10.1371/journal.pone.0045370

**Published:** 2012-09-25

**Authors:** Kieran C. R. Fox, Pierre Zakarauskas, Matt Dixon, Melissa Ellamil, Evan Thompson, Kalina Christoff

**Affiliations:** 1 Department of Psychology, University of British Columbia, Vancouver, British Columbia, Canada; 2 Brain Research Centre, University of British Columbia, Vancouver, British Columbia, Canada; 3 Department of Philosophy, University of Toronto, Toronto, Ontario, Canada; CSIC-Univ Miguel Hernandez, Spain

## Abstract

The accuracy of subjective reports, especially those involving introspection of one's own internal processes, remains unclear, and research has demonstrated large individual differences in introspective accuracy. It has been hypothesized that introspective accuracy may be heightened in persons who engage in meditation practices, due to the highly introspective nature of such practices. We undertook a preliminary exploration of this hypothesis, examining introspective accuracy in a cross-section of meditation practitioners (1–15,000 hrs experience). Introspective accuracy was assessed by comparing subjective reports of tactile sensitivity for each of 20 body regions during a ‘body-scanning’ meditation with averaged, objective measures of tactile sensitivity (mean size of body representation area in primary somatosensory cortex; two-point discrimination threshold) as reported in prior research. Expert meditators showed significantly better introspective accuracy than novices; overall meditation experience also significantly predicted individual introspective accuracy. These results suggest that long-term meditators provide more accurate introspective reports than novices.

## Introduction

William James exhorted us more than a century ago, “Introspective observation is what we have to rely on first and foremost and always” [1], but for much of the 20^th^ century, psychologists did not regard introspective reports as valid data for scientific inquiry. Some contemporary researchers have doubted the very possibility of accurate introspection [2]; others have demonstrated that while introspective reports may be reliable under simple conditions, reliability decreases with increasing demands on central processing resources [3].

Introspection can of course be defined in many ways; here we mean it in the straightforward manner used by James: “The word introspection need hardly be defined – it means, of course, looking into our own minds” [1]. That is, in its simplest form introspection involves “considerations of our own experience… [and] our own internal states” [4].

‘Introspective accuracy’ (IA) can putatively be quantified by a variety of methods that combine introspective reports of subjective, mental phenomena with some objective (neural, physiological, or behavioral) measure of these same phenomena. A subject's IA with respect to a given task or process is the degree to which their introspective reports agree or correlate with such objective measures [3], [5].

Recent research provides evidence for large inter-individual variability in introspective accuracy, which may be traceable to and predicted by differential grey matter volume in rostrolateral prefrontal cortex (RLPFC)/Brodmann Area (BA) 10 [5]. Individual differences with respect to a given skill invite the question of whether that skill can be ameliorated, and a recent study involving extensive training supports the idea that well-trained subjects can provide accurate and useful introspective reports [6] (though direct improvement of introspection through training has yet to be demonstrated, to our knowledge). Further, RLPFC/BA10, thought to be a key region involved in introspection and metacognitive awareness [7], is amenable to voluntary up- and down-regulation through real-time functional magnetic resonance imaging (fMRI) neurofeedback training [8]. This functional plasticity [8] and structural heterogeneity [5] in frontal regions key to introspection thus provides a possible neural basis for inter-individual differences, and possibly intra-individual enhancements, in introspective accuracy.

In parallel with this renewed interest in introspection, cognitive neuroscience has begun to focus on the family of mental training practices known as ‘meditation’ [9]. Many meditation practices are highly introspective in nature: common techniques direct the meditator's attention toward emotional states, the arising of thoughts, and even the quality and focus of attention itself [10], [12], [13]. This heavy focus on introspection has led to the hypothesis that experienced meditators might possess the capacity for more objective assessment of their own internal states and mental contents (i.e., greater introspective accuracy) [10], [11]. While a recent study examining subjective reports of emotional state alongside objective measures of autonomic arousal found that long-term meditators' introspective reports correlated better with objective measures than did reports from meditation-naïve controls [14], other similar work has shown equivocal results [15], or no differences between meditators and controls [16]. The evidence for enhanced introspective accuracy in long-term meditators, then, remains meager.

One particular meditation technique, *vipassana* (‘Insight’) meditation (VM), includes paying close attention to the inner experiences (conceptual, emotional, tactile, and visceral) associated with the current state of the body, primarily in order to better develop a non-discursive awareness centered in the present moment [12], [13]. Such practices may involve the meta-representation by the brain of diverse internal bodily responses and states [10], a view supported by a number of neuroimaging studies of VM meditators, as well as subjects engaging in Mindfulness-Based Stress Reduction (MBSR) courses (at heart a secularized version of VM, with a comparable focus on breath sensations, body awareness, etc. [12]). Neuroimaging has shown that among VM and MBSR meditators the insula, a region whose grey matter volume predicts the accuracy of interoceptive reports [17], exhibits increased cortical thickness [18] and grey matter density [19], as well as increased fMRI blood oxygen-level dependent (BOLD) signal during present-centered awareness [20]. VM and MBSR meditators also show structural and functional augmentations of primary and secondary somatosensory cortices, including increased cortical thickness [18] and fMRI-BOLD signal [20]. Finally, VM meditators show significantly thicker cortex [18] (and in Tibetan Buddhist practitioners, increased grey matter density [22]) in RLPFC/BA10, suggesting enhancement of a region strongly implicated in introspection [5], [7], [8], [45].

Despite this converging evidence that introspection and body-awareness may be heightened in VM/Mindfulness meditators, and despite the extensive body of objective data on tactile sensitivity in humans with which subjective reports could be compared, no study has yet examined the accuracy of introspective reports from a representative cross-sectional group of VM practitioners.

VM provides an ideal means of exploring introspective accuracy: the body-scanning meditation (BSM; *vedananupassana*) practice within this tradition focuses intensively on awareness of ambient tactile experiences of an entirely subjective nature, varying greatly in quality and intensity. Complementary scientific exploration of tactile sensibility has been extensive in humans, and has likewise shown marked variability in regional sensitivity throughout the body. Correlating subjective with objective measures of tactile sensitivity can thus provide a convenient measure of the extent to which introspective reports agree with what is to be expected from neurophysiological measures.

To explore this idea, we first gathered two sets of well-replicated, objective data on tactile sensitivity from previously published research that involved large samples of adults: (i) psychophysical discrimination and (ii) proportion of cortical area dedicated to various body regions in primary somatosensory cortex (S1).

In his treatise *De Tactu*, Ernst Weber [23] established the now-classic two-point discrimination (2PD) task as a basic psychophysical measure, documenting the differential sensitivity of the sense of touch throughout the body (replicated by Weinstein [24]). Improved neurosurgical methods later allowed direct electrophysiological exploration of S1 in humans, resulting in the famous ‘sensory homunculus’ illustrating the differential cortical representation of body regions [25], [26]. The patterns of psychophysical sensitivity and cortical area are closely correlated (*r* = .65); i.e., regions of the body more sensitive by psychophysical measures tend to have a greater area of S1 dedicated to them [24] (Table 1).

**Table 1:** Psychophysical and cortical measures of tactile sensitivity throughout the body.

2PD threshold (rank)^a,b^	Adjusted Area of S1 Cortex (rank)^b,c,d^
Middle Finger (20)	Lips (20)
Index Finger (19)	Nose (19)
Thumb (18)	Thumb (18)
Ring Finger (17)	Little Finger (17)
Little Finger (16)	Sole (16)
Lips (15)	Ring Finger (15)
Cheek (14)	Middle Finger (14)
Nose (13)	Index Finger (13)
Palm (12)	Big toe (12)
Big toe (11)	Forehead (11)
Forehead (10)	Cheek (10)
Sole (9)	Calf (9)
Abdomen (8)	Upper Arm (8)
Chest (7)	Forearm (7)
Forearm (6)	Thigh (6)
Shoulder (5)	Back (5)
Back (4)	Shoulder (4)
Upper Arm (3)	Palm (3)
Thigh (2)	Chest (2)
Calf (1)	Abdomen (1)

Reverse rank-ordered tactile sensitivity for each of the twenty body regions examined, according to psychophysical (2PD threshold) and cortical (area of S1, adjusted for corresponding skin surface area) measures, as reported in previous research. Psychophysical and cortical measures were strongly correlated [*r*(19) = .65, *p* = .002]. ^a^[Ref. 23]; ^b^[Ref. 24]; ^c^[Ref. 25]; ^d^[Ref. 26] (esp. Fig. 17, pg. 44). 2PD: two-point discrimination; S1: primary somatosensory cortex.

VM instructors teaching BSM assert that even while sitting quietly, *without* overt tactile stimulation, attention can nonetheless be turned to the conceptual, emotional, tactile and visceral experiences related to the present state of the body, and that the experiences that arise will likewise vary in intensity across body regions [13]. During BSM, practitioners focus their awareness progressively on every point of the body's surface, waiting until an experience of some kind arises and calmly registering its occurrence. Certain areas (e.g., fingertips, face) tend to yield very clear, intense experiences, while others (e.g., back, legs) tend to be more dull and undifferentiated [13].

In order to test the ‘neurophenomenology’ hypothesis [11] that self-reports will correlate better with objective measures in individuals with contemplative training [10], we collected subjective reports of tactile experiences during a session of BSM from meditators with a broad, representative cross-section of experience (1–15,000 hrs experience) and compared them to objective neural and psychophysical measures.

## Materials and Methods

### Participants

A total of 42 meditation practitioners (‘meditators’) participated. Four participants' data were dropped due to noncompliance with instructions (e.g., circling more than one answer on the sensitivity scale), leaving a total of 38 participants (19 female; mean age  = 41.7±16.1 years). All participants had prior experience with and interest in Insight meditation (mean time since beginning meditation practice  = 11.0±10.3 yrs), though overall hours of experience (MED) varied enormously (*M* = 2051±3600 hrs; *min*.  = 1.0 hrs; *max*.  = 15,000 hrs). The range of experience with the BSM practice in particular, though not as extensive, also varied greatly (*M* = 154±322 hrs; *min*.  = 0 hrs; *max*.  = 1643 hrs), and was correlated with MED [*r*(37) = .36, *p* = .025]. Participants were recruited through the UBC Meditation Community, the B.C. Insight Meditation Society, and referrals. The University of British Columbia Behavioral Research Ethics Board approved the study protocol. Participants provided written informed consent and were debriefed at the end of the experiment.

### Procedure

We led a cross-sectional group of meditators (1 hr–15,000 hrs of experience) through a session of BSM [13] for approximately 30 minutes, and immediately afterward collected their subjective reports on the sensitivity of 20 regions throughout the body. These subjective scores were then correlated with objective psychophysical and cortical measures of tactile sensitivity gleaned from previous research (described below). Most participants (*n* = 30), including all novices, were led through the BSM session by a highly experienced meditation instructor. A few (*n* = 8) highly experienced meditators (300–15,000 hrs experience), who had previously received detailed instruction in BSM from a qualified instructor (via intensive retreats of 10+ days) were permitted to practice BSM independently. 33 of 38 participants had at least some prior experience with BSM. Following the meditation, participants completed a questionnaire about their subjective experiences during the BSM session (see below); finally, participants filled out a brief biographical questionnaire.

### Objective Measures of Sensitivity

#### Psychophysical Measure

Average values for two-point discrimination (2PD) thresholds for each of 20 body regions, as reported in previous research, were used. Data are from 48 participants (mean age  = 22 years; 24 female) [24]. The 2PD task measures the minimal interstimulus distance required to perceive two simultaneously applied stimuli as distinct [23] with the regional sensitivity of the skin varying markedly [24] (Table 1). While data from Weinstein [24] are used here because they are the most comprehensive (20 regions tested), a recent systematic study (13 regions, *n* = 122) has largely replicated his results [40]; other recent work (testing fewer body regions) also shows comparable discrimination thresholds (e.g., [41]).

#### Cortical Measure

Average values for total area in primary somatosensory cortex (S1) for 20 body regions were likewise gleaned from prior published research. Rank-order values for average total area of cortex in S1 dedicated to a given body region were used. Data are based on [25] and [26]; rank-orderings for cortical area adjusted for skin surface area (ACA: adjusted cortical area; Table 1) follow [24]. Cortical data represent aggregated sensation reports from 126 patients undergoing neurosurgery involving direct galvanic stimulation of S1 [25]. Further electrophysiological explorations of S1 have replicated the work of Penfield and colleagues [25], [26], with minor modifications [27], [28]. Parallel explorations of S1 with magnetoencephalography [29] and fMRI in both humans [30] and monkeys [31] further support the results of Penfield and colleagues.

#### Composite Somatic Sensitivity Rank (SSR)

As the objective measures (2PD and ACA) were found to be highly correlated (*r* = .65, *p* = .002), a composite Somatic Sensitivity Rank (SSR) was calculated by averaging the rankings from both measures for each body region. The SSR represents a mixed psychophysical-cortical measure of somatic sensitivity, and provided a convenient single measure of ‘somatic’ sensitivity for a given body region.

### Subjective Measure of Sensitivity

#### Sensory Sensitivity Survey

**Figure 1:**
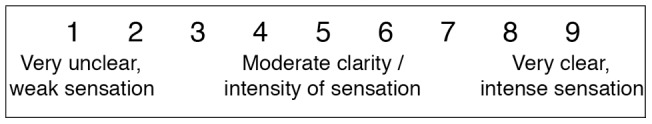
Subjective sensitivity scale used for self-reported sensitivity of 20 body regions after BSM session.

**Figure 2:**
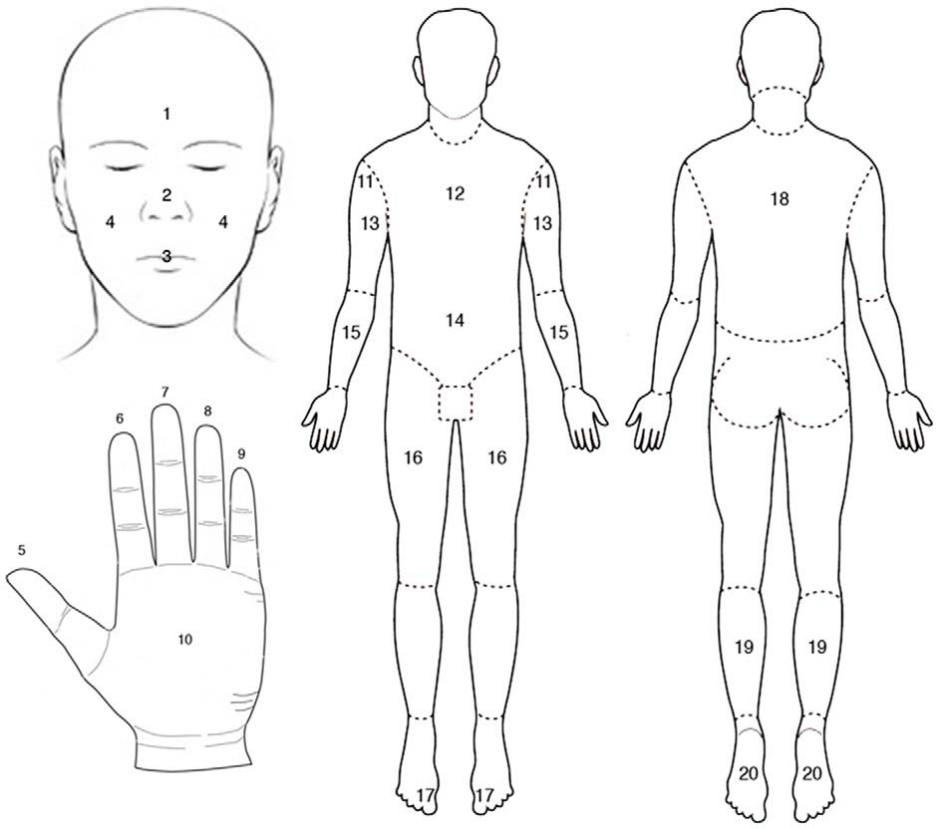
Line diagrams used in the Subjective Sensory Sensitivity questionnaire. Participants provided a rating (on a scale of 1–9; Fig. 1) of the relative, subjective sensitivity of each region during their meditation experience. The 20 regions were simply numbered from top to bottom and front to back of the body; this pattern of numbering bore no relation to the rankings of objective sensitivity measures with which subjective reports were compared. All body regions listed in Table 1.

Following the session of BSM, participants silently and individually filled out a survey of their subjective experiences during the meditation. Detailed instructions were provided and the experimenter was available to resolve any difficulties. The survey showed diagrams of the body alongside a simple scale (Fig. 1 and Fig. 2) asking for the “clarity and/or intensity” of sensations in each region *relative* to each other region. Obvious differences in ‘body-awareness’ were obviated by requiring use of the full range of the scale, such that even highly experienced practitioners rated *some* region(s) ‘1’ (lowest sensitivity *for them*). Similarly, novices rated as ‘9’ the region(s) with the highest sensitivity *for them*. Thus the survey required participants to introspect on and evaluate the relative intensity of their experiences during BSM, evaluating *relative* differential clarity/intensity of experience for each of the 20 regions, regardless of *absolute* clarity or intensity (mean ± SD subjective scores for all body regions, for all subjects: Table S2).

#### Measuring Expertise

A major methodological question is how to measure ‘experience’ or ‘expertise’ in the context of meditative training. Here meditators reported overall hours of meditation experience in general, and BSM in particular. When examining a wide range of experience with respect to a particular skill, achievement is typically related to practice time logarithmically [32]. Such nonlinear relationships between achievement and practice time, suggestive of diminishing returns with invested practice, have been demonstrated for an enormous variety of mental and physical skills [32], [33], including possibly meditation [34]. We observed a comparable effect here, where hours of experience and introspective accuracy exhibited a log-linear relationship (see Results). As most participants (36/38) provided a precise date when they first began meditating, we also derived a rough measure of ‘practice intensity’ (PI) by dividing total hours of meditation experience by number of months since beginning meditation practice for each participant. This resulted in an average number of hours spent in meditation per month over each participant's meditation career. Introspective accuracy for all objective measures was further correlated with PI.

### Data Analysis

#### Calculating Individual Introspective Accuracy

Sensitivity scores for each of the 20 body regions were collected from participants and then correlated with psychophysical (2PD), cortical (ACA) and composite (SSR) measures, resulting in three correlation scores for each subject. Higher psychophysical discriminative capacity is represented by smaller interstimulus distances in mm, and higher cortical area rank is likewise represented by smaller values, whereas in the subjective sensitivity scale used (Fig. 1), high values represent high sensitivity for a given body area. Thus, for the sake of clarity, all objective measures were reverse rank-ordered (Table 1), so that strong positive correlations represent a close fit between subjective and objective measures, or higher Introspective Accuracy (IA).

#### Novices Contrasted with Experts

To explicitly contrast novice and expert meditators without setting an arbitrary hours-of-experience threshold for either group, the sample was divided into quartiles by overall hours of meditation experience (MED), and the bottom and top quartiles were used to assign ‘novice’ and ‘expert’ subgroups, respectively. This division relied solely on the assumption that those meditators with the most experience would differ from those in our sample with the least experience, and was therefore blind to actual individual introspective accuracy. Within our sample of 38 meditators, this resulted in two groups of nine participants each: MED-Experts (*n* = 9, mean MED  = 7231±4410 hrs; 6 male; mean age  = 50±18 yrs) and MED-Novices (*n* = 9, mean MED  = 28±24 hrs; 6 female; mean age  = 29±8 yrs). We similarly divided our sample into upper and lower quartiles by BSM experience (again, blind to IA scores), creating BSM-Expert (*n* = 9, mean BSM  = 571±469 hrs; 6 males; mean age  = 43±*17* yrs) and BSM-Novice (*n* = 9, mean BSM  = 0.40±0.55 hrs; 5 males; mean age  = 43±*19*) groups.

#### Mean Introspective Accuracy for Experts and Novices

As a complementary test of Expert-Novice differences, we directly calculated mean introspective accuracy for each MED subgroup, by averaging the raw subjective scores on the Sensory Sensitivity Study for MED-Experts and MED-Novices. This resulted in mean subjective sensitivity scores for each body region, for both Experts and Novices. Both sets of mean subjective scores were correlated with SSR to obtain mean introspective accuracy scores for each subgroup.

#### Introspective Accuracy as Predicted by Meditation Experience

Taking the entire sample as a whole (not merely Experts and Novices), we correlated each subject's introspective accuracy score with (i) their amount of experience with BSM and (ii) their overall meditation experience, in order to investigate whether amount of BSM practice in particular, or meditation practice generally, predicts introspective accuracy. As scatterplots (not shown) showed logarithmic relationships and strong positive (right) skewness (suggestive of diminishing returns on invested practice, and highly reminiscent of many skill-learning curves) the natural logarithm (ln) of hours of experience for both MED and BSM was calculated for each subject, and correlated with their introspective accuracy.

## Results

### Novices Contrasted with Experts

We first contrasted experts and novice meditators (the bottom and top quartiles of our sample in terms of overall meditation experience (MED) – see Methods). Individual subject correlations (between subjects' subjective reports and each objective measure) were Fisher-transformed [35] and averaged within groups to obtain mean, group correlations for each objective measure. The two groups' means were compared with independent samples *t*-tests, which showed significant differences between MED-Experts and MED-Novices across all measures, thus suggesting more accurate introspective reports in MED-Experts as compared to MED-Novices (Table 2). We similarly divided our sample into upper and lower quartiles by BSM experience (again, blind to IA scores – see Methods) and compared introspective accuracy across groups. Independent samples *t*-tests comparing BSM-Experts and BSM-Novices showed significantly greater average (Fisher-transformed) correlations for BSM-Experts on all measures (Table 3); moreoever, effect sizes were larger than when comparing MED-Experts and MED-Novices.

**Table 2:** Average correlations with each objective measure for MED-Expert and MED-Novice meditators.

Objective Measure	MED-Experts (*n* = 9)	MED-Novices (*n* = 9)	Comparison of mean *r*'s	Effect size (Cohen's *d*)
2-Point Discrimination	mean *r* = .46	mean *r* = −.01	*t*(16) = 2.229, *p* = .041	1.12
Adjusted Cortical Area	mean *r* = .31	mean *r* = −.16	*t*(16) = 2.677, *p* = .017	1.31
Somatic Sensitivity Rank	mean *r* = .44	mean *r* = −.11	*t*(16) = 2.787, *p* = .013	1.39

**Table 3:** Average correlations with each objective measure for BSM-Expert and BSM-Novice meditators.

Objective Measure	BSM-Experts (*n* = 9)	BSM-Novices (*n* = 9)	Comparison of mean *r*'s	Effect size (Cohen's *d*)
2-Point Discrimination	mean *r* = .64	mean *r* = .18	*t*(16) = 3.004, *p* = .008	1.51
Adjusted Cortical Area	mean *r* = .41	mean *r* = .06	*t*(16) = 3.365, *p* = .004	1.69
Somatic Sensitivity Rank	mean *r* = .58	mean *r* = .12	*t*(16) = 3.134, *p* = .006	1.57

### Mean Introspective Accuracy for Experts and Novices

We also calculated mean, group introspective accuracy for both MED-Experts and MED-Novices. Experts' averaged subjective sensitivity scores were found to correlate highly significantly with SSR (*r* = .87, *p*<.001) (Table 4), indicating extremely high mean introspective accuracy among MED-Experts. Conversely, Novices' averaged subjective sensitivity scores did not correlate significantly with SSR (*r* = –.23, n.s.) (Table 4), suggesting very poor introspective accuracy as a group for MED-Novices.

**Table 4:** Introspective Accuracy compared between groups of Experts and Novices.

Somatic Sensitivity Rank (2PD+ACA)	Novices' Sensitivity Score (mean; 1–9)	Experts' Sensitivity Score (mean; 1–9)
Thumb (20)	Back (6.3)	Lips (7.4)
Lips (19)	Abdomen (5.9)	Thumb (7.4)
Middle Finger (18)	Chest (5.8)	Index Finger (7.2)
Little Finger (17)	Lips (5.4)	Middle Finger (7.0)
Index Finger (16)	Palm (5.0)	Little Finger (7.0)
Nose (15)	Thumb (5.0)	Palm (6.8)
Ring Finger (14)	Thigh (4.9)	Nose (6.7)
Sole (13)	Forehead (4.9)	Ring Finger (6.3)
Cheek (12)	Shoulder (4.8)	Sole (6.2)
Big toe (11)	Nose (4.8)	Big toe (6.1)
Forehead(10)	Index Finger (4.7)	Forehead (6.0)
Palm (9)	Cheek (4.4)	Abdomen (6.0)
Forearm (8)	Middle Finger (4.1)	Cheek (5.7)
Upper Arm (7)	Little Finger (3.9)	Chest (5.4)
Calf (6)	Ring Finger (3.7)	Shoulder (5.0)
Abdomen (5)	Forearm (3.6)	Back (5.0)
Chest (4)	Sole (3.6)	Forearm (4.8)
Shoulder (3)	Calf (3.4)	Upper Arm(4.7)
Back (2)	Big toe (3.4)	Thigh (4.6)
Thigh (1)	Upper Arm (3.1)	Calf (4.4)

Composite Somatic Sensitivity Rank for the 20 body regions assessed alongside averaged sensitivity scores for MED-Novice and MED-Expert meditators for each body region (on a 1–9 scale). Experts' mean subjective scores correlated strongly and significantly with SSR, whereas Novices' mean scores correlated negatively and nonsignificantly.

### Introspective Accuracy as Predicted by Body-Scanning Meditation (BSM) Experience

The total number of hours previously spent in body-scanning meditation (BSM) significantly predicted the relationship between first-person sensitivity reports and all objective measures (Table 5); the natural logarithm (ln) of hours of BSM experience (logBSM) also significantly predicted all relationships (Table 5), even after using partial correlations to control for age (Table S1). As we predicted *a priori* that meditators with more BSM experience would have improved correlations at the individual level, one-tailed tests were used for these analyses.

**Table 5:** Introspective accuracy as predicted by various measures of meditation expertise.

	Introspective Accuracy Correlated with Measure of Meditation Experience			
Objective Measure	MED	logMED	BSM	logBSM
2-Point Discrimination	*r*(37) = .33, *p* = .046	*r*(37) = .37, *p* = .024	*r*(37) = .39, *p* = .009, one-tailed	*r*(37) = .34, *p* = .019, one-tailed
Adjusted Cortical Area	*r*(37) = .38, *p* = .020	*r*(37) = .48, *p* = .003	*r*(37) = .32, *p* = .026, one-tailed	*r*(37) = .32, *p* = .026, one-tailed
Somatic Sensitivity Rank	*r*(37) = .39, *p* = .019	*r*(37) = .45, *p* = .006	*r*(37) = .39, *p* = .009, one-tailed	*r*(37) = .36, *p* = .014, one-tailed

Correlations between sensitivity as ranked by various physiological measures and as ranked by subjective reports, regressed on overall meditation experience (MED) or BSM experience, or their log values. Significant correlations here show that individual introspective accuracy improves with increasing meditation experience. Tests are two-tailed unless otherwise indicated.

### Introspective Accuracy as Predicted by Overall Meditation Experience (MED)

**Figure 3:**
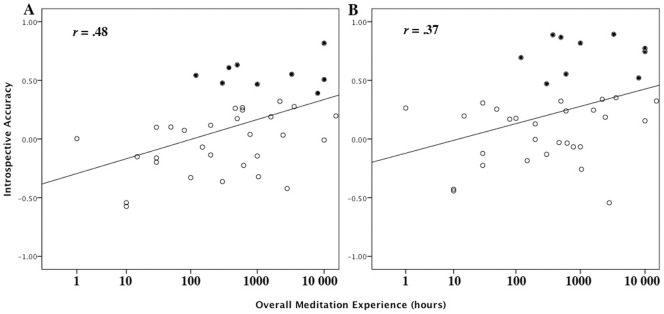
Introspective accuracy (individual correlations between subjective sensitivity reports and adjusted area of primary somatosensory cortex [A] and 2-point discrimination threshold [B]) as a function of overall meditation experience. Filled data points indicate practitioners whose introspective accuracy correlations were significant at the individual level (*p*<.05, one-tailed).

Overall hours spent in all forms of meditation combined (MED), which included hours spent in BSM, significantly predicted the relationship between subjective sensitivity scores and all objective measures (Table 5). Relationships with all objective measures were significantly (indeed, better) predicted by logMED (Table 5; Fig. 3A, 3B), even when using partial correlations to control for age (Table S1).

### Introspective Accuracy when Controlling for BSM Experience

As we found that MED strongly predicted IA (even better than hours of BSM practice), we suspected that long-term meditators might be showing generalized enhancement of Introspective Accuracy, independent of BSM experience. We reasoned that, over and above experience with BSM, clearly useful for the task at hand, meditation experience generally might predict IA (even when measuring IA with a task so heavily dependent on body awareness). To test this hypothesis, we subtracted total hours of BSM experience from overall hours of meditation (MED), resulting in a measure of total *other* meditation experience (MED_other_). We again took the natural logarithm of MED_other_, expecting it to similarly be a better predictor than raw hours (logMED_other_). Controlling for the effect of BSM, we found that logMED_other_ significantly predicted IA for ACA [Partial *r*(35) = .36, *p* = .014] and SSR [Partial *r*(35) = .31, *p* = .032], with a trend toward predicting IA for 2PD [Partial *r*(35) = .22, *p* = .096], indicating that, on balance, other meditation experience does predict IA even on a body awareness-related task, over and above number of hours spent training in BSM specifically. Even after further controlling for Age, partial correlations remained significant for ACA [Partial *r*(34) = .31, *p* = .032], and nearly held for SSR [Partial *r*(34) = .28, *p* = .051]. (As we predicted the ancillary effect of logMED_other_ *a priori*, all tests here were one-tailed).

### Introspective Accuracy as Predicted by Practice Intensity (PI)

Practice Intensity (mean hours meditating per month over each participant's meditation career) also significantly predicted IA for all measures: PI with 2PD, [*r*(35) = .36, *p* = .034]; PI with ACA, [*r*(35) = .45, *p* = .007]; PI with SSR, [*r*(35) = .43, *p* = .009].

### Age and Gender

Neither age (*M±SD* = 41.1±16.1 yrs) nor gender (19 female, 19 male) significantly predicted individual correlations with any of the objective measures used (for age: all *r*'s <.19, all *p*'s >.27; for gender: all *r*'s <.10, all *p*'s >.58).

## Discussion

Not long ago, Nisbett and Wilson [2] argued that “the accuracy of subjective reports is so poor as to suggest than any introspective access that may exist is not sufficient to produce generally correct or reliable reports” (p. 233). Our results both support and contrast with this conclusion, suggesting that untrained persons indeed have very poor introspective accuracy, but that this skill might be improved with training. We found that in highly experienced *vipassana* meditators, subjective reports of the clarity and/or intensity of tactile experiences during a body-scan meditation correlated significantly as a group (and often at the individual level) with two objective measures of sensitivity gathered from prior published research, as well as with a composite measure combining psychophysical and cortical data. Novice meditators, in contrast, did not show significant correlations at the group or individual level with any measure. Pooling all subjects, we found that overall meditation experience, overall BSM experience, and Practice Intensity all significantly predicted individual introspective accuracy on all measures, suggesting that not only overall experience, but also the ardor of meditation practice may contribute significantly to introspective accuracy. To our knowledge, this represents the first study to investigate a continuous and representative cross-section (novices to experts, with experience spanning 15,000 hrs) of meditation experience, yielding results showing that meditation experience (hours of practice) significantly predicts introspective accuracy in a ‘dose-dependent’ fashion.

Though subject to wide variability, the general trend suggests that with increasing meditation experience, reports of subjective tactile experience are more and more closely aligned with what would be expected from a purely neurophysiological perspective. The simplest interpretation of these results is that subjects with greater meditation experience may provide more accurate reports of mental experience.

An alternative explanation is that the perceptual acuity of VM meditators has been heightened in the tactile modality (enhanced visual [36] and tactile [39] acuity have both been found after intensive meditation practice). On this view, long-term meditators would be neither better introspectors on, nor more accurate reporters of, inner experience, but simply more perceptive than novices.

We consider this an unlikely explanation of our results, however, for several reasons: (i) no overt tactile stimulation was actually present in our study. The origin of the sensations subjectively experienced during BSM may be peripheral (arising from activity at the skin's mechanoreceptors, or peripheral nerves), central (arising from activity in somatosensory regions of the brain), or some combination of both. In any case, whatever meditators are reporting on, it does not involve perceptual discrimination of explicit sensory stimulation, as in the study of visual acuity [36] or the standard 2PD task [23]; (ii) reports were retrospective: even if all experiences arose peripherally (e.g., at the mechanoreceptors of the skin), meditators were reporting on second-order representations of these experiences held in memory, rather than making real-time perceptual judgments; (iii) reports were evaluative: meditators were specifically instructed to evaluate their overall experience during the BSM session and give *relative* ratings of intensity for each region, rather than simply reporting the intensity for a given body region. The subjective reports, therefore, do not constitute reports of sensory experience, but rather judgments of the clarity of various (recalled) internal experiences relative to one another; (iv) it is tempting to assume that sensations subjectively ‘felt’ throughout the body at the level of the skin would bear a closer relation to peripheral than central nervous system activity (and hence best considered perceptual or sensory, rather than strictly ‘mental,’ experiences). The first study to test this hypothesis in humans, however, found no evidence for a direct relationship between intensity of sensation as measured by subjective reports versus by density of action potentials in the median and ulnar nerves [38]. The authors concluded that cortical areas were thus far more likely to play the central role in generating subjective tactile experience, *even during overt tactile stimulation* [38]. During BSM, where no overt tactile stimulation is present, this seems all the more likely – inconsistent with the notion that enhanced accuracy of reports is due solely to improvements in low-level sensory acuity. So while a heightening of perceptual acuity may indeed result from long practice of BSM, and may support or interact with introspection on experiences during meditation, we believe that the enhancement of introspective accuracy remains the most parsimonious explanation of our results.

Numerous limitations must be taken into consideration, however – most notably the use of averaged psychophysical and cortical measures gleaned from the literature. Though we used data from large samples [24], [25] that have been replicated repeatedly [27], [28], [40], [41], and so ought to be generalizable to the population at large (see Methods), there is no substitute for individual psychophysical testing or cortical mapping. Thus a key question that cannot be answered by the present study concerns the relationship between subjective reports and an individual's objective measures of tactile sensitivity. Future work could examine this relationship by performing extensive psychophysical testing on individual meditators, or by using neuroimaging to compare subjective reports with the morphology of key interoceptive (insula) and exteroceptive (somatosensory) areas of the brain already known to be enhanced in expert meditators [18], [19], [21].

The cross-sectional nature of our sample of meditators precludes inferring a direct causal link between meditation practice and greater introspective accuracy. Though experience level strongly predicted introspective accuracy at the individual level, it may be that practitioners who persist in a long-term meditation practice already begin with higher introspective accuracy; further work could experimentally examine possible training effects from BSM using a pre-post design along with a suitable (e.g., wait-list) control group. We did observe, however, a logarithmic relationship between experience level and IA, suggestive of diminishing returns on accuracy with increasing practice time – a trend strongly reminiscent of many forms of skill learning [33] – and though not all expert meditators demonstrated high introspective accuracy, *no* novice meditators did. Further exploration of potential meditation training effects using experimental (rather than cross-sectional) designs therefore seems warranted.

We agree with others [9] in conceptualizing meditation as a form of ‘mental training’ – as such, it stands to reason that meditation training will be subject to the same benefits (improved performance) and constraints (diminishing returns) observed nearly ubiquitously among many forms of mental and physical skill learning [32], [33]. Unlike many skills, however, we found evidence that the introspective skills of experienced meditators may be generalizable. Unexpectedly, overall meditation experience was a better predictor of introspective accuracy than total BSM experience (Table 5); further, we found strong (though not definitive) evidence that total meditation experience, over and above BSM experience, still significantly predicted Introspective Accuracy. These results are consistent with research showing generalizable improvements in perceptual discrimination and sustained attention [36], as well as visuospatial processing [37], in experienced meditators, and suggest that enhanced introspective accuracy may be generalizable to multiple domains.

The potential mechanisms of enhanced introspective accuracy remain an intriguing issue. Inter-subject differences in introspective accuracy are predicted by grey matter volume in RLPFC/BA10 [5], and within subjects, the practice of introspection itself modulates activity in this same region [8]. If intensive introspective practice during meditation recruits RLPFC/BA10, use-dependent structural or functional alterations there might explain the enhancement of introspective accuracy – and as noted above, two studies have already reported structural differences in this region in long-term meditators [18], [22]. Considering the specific nature of the meditation engaged in by BSM practitioners, we consider plasticity in cortical regions related to body awareness (e.g., S1, insula) another important means whereby introspective accuracy for tactile sensitivity might be improved. Use-dependent plasticity is well known in somatosensory brain regions [42], [43], and the grey matter volume of the insula has been shown to predict the accuracy of interoceptive reports [17]. As noted earlier, alteration of structure and/or function has been demonstrated in both these regions in long-term meditators [18]–[21]; such changes may mediate enhanced body awareness.

Other forms of mental practice or training might also lead to similar results. Intriguingly, a recent study examining spontaneous (ambient) sensations in healthy normal subjects found that heightened attention increased the intensity of the subjective tactile experiences [47]. Though examining ambient sensations in the hands only, the quantity of spontaneous sensation reports followed a proximo-distal gradient reminiscent of psychophysical measures and mechanoreceptor density in the hand, paralleling our results here. Multiple sessions and an elaborate response protocol were required, however, to elicit reports in line with physiological measures, and only one region of the body was tested – whereas with the expert meditators in our study, high subjective-objective measure correlations were obtained for 20 body regions probed only once each. Nonetheless, the results of Michael and Naveteur [47] suggest (i) that non-meditators too can become aware of, and eventually report objectively on, spontaneous tactile experiences after a few sessions of practice; and (ii) that the ambient sensations experienced by BSM meditators are not simply an artifact of their meditation technique, but a normal physiological phenomenon – one they simply pay more attention to than other people.

How might body awareness and introspection interact? As BSM involves the intensive, simultaneous practice of both awareness of the body, as well as introspection on thoughts and emotions, a kind of Hebbian learning may take place: the concentrated, frequent coupling of dispassionate introspection with attention to the body may enhance introspective accuracy for internal experiences related to the body especially, as well as for mental events generally. That is, even as more (or more detailed) information about the state of the body is reaching awareness, the objectivity with which experience is evaluated increases, resulting in an enhanced, more objective awareness of the body and the mental events related to its momentary experience. Conversely, practitioners who engage in non-BSM forms of meditation may enhance introspection in particular, which generalizes to an enhanced awareness of the body. Supporting this possibility, a recent study found that individual introspective accuracy is stable across multiple different perceptual tasks, and may thus relie on a relatively general system [44]; if so, *enhancements* of introspective accuracy, too, may ameliorate a single multi-purpose system, leading to generalizable improvements – a view consistent with the claims advanced by meditation practitioners that “the development of awareness with any particular meditation technique will automatically result in a marked increase in one's general level of awareness, thereby enhancing one's capacity to be mindful in regard to situations that do not form part of one's primary object of meditation” ([46]; p.22]). Pending further study, however, views on the potential mechanisms whereby introspective accuracy might be enhanced and/or generalizable to other domains remain speculative. Clearly, much research remains to be done in this nascent area.

In summary, we found that introspection, as measured by subjective assessments of tactile experiences during meditation, becomes more accurate with increasing meditation experience. If this improved introspective accuracy can be generalized to other domains, then experienced meditators may prove to be powerful collaborators for cognitive neuroscientists exploring the neural correlates of higher cognition, abstract thought, and consciousness [11]. Our findings are consistent with studies of experienced meditators showing enhancement of structure and/or function in areas key to interoception (insula), exteroception (somatosensory cortices), and introspection generally (RLPFC/BA10). Further research investigating introspective accuracy in meditators seems warranted, specifically with the aims of (i) determining whether meditation training plays a causal role in the differences observed between novices and experts; (ii) elucidating the individual neurophysiological basis for such differences; and (iii) assessing whether enhanced introspective accuracy is specific to the domain in which it was developed, or can be generalized to others.

## Supporting Information

Table S1 **Partial correlations (controlling for age) between Introspective Accuracy with various physiological measures and overall meditation experience (MED) or BSM experience.** Significant correlations here indicate that individual introspective accuracy improves with increasing meditation experience, even when age is controlled for.
(DOC)

Table S2 **Mean subjective sensitivity scores (±** ***SD*** **) for each of the 20 body regions tested, averaged across all participants.** IF: index finger; MF: middle finger; LF: little finger; RF: ring finger.
(DOC)
