# Supplementary material for: Meditation Experience Predicts Introspective Accuracy
Source: PLoS One. 2012 Sep 25;7(9):e45370. doi: 10.1371/journal.pone.0045370 (PMC3458044; doi:10.1371/journal.pone.0045370)
Supplement: Table S1 — Partial correlations (controlling for age) between Introspective Accuracy with various physiological measures and overall meditation experience (MED) or BSM experience. Significant correlations here indicate that individual introspective accuracy improves with increasing meditation experience, even when age is controlled for. (DOC) [file pone.0045370.s001.doc]

| **Objective Measure** | **Introspective Accuracy correlated with logMED** | **Introspective Accuracy correlated with logBSM** |
| --- | --- | --- |
| 2-Point Discrimination | Partial *r*(35) = .36, *p* = .028 | Partial *r*(35) = .35, *p* = .036 |
| Adjusted Cortical Area | Partial *r*(35) = .45, *p* = .004 | Partial *r*(35) = .33, *p* =. 048 |
| Somatic Sensitivity Rank | Partial *r*(35) = .44, *p* = .006 | Partial *r*(35) = .37, *p* = .024 |
