# Supplementary material for: Meditation Experience Predicts Introspective Accuracy
Source: PLoS One. 2012 Sep 25;7(9):e45370. doi: 10.1371/journal.pone.0045370 (PMC3458044; doi:10.1371/journal.pone.0045370)
Supplement: Table S2 — Mean subjective sensitivity scores (± SD ) for each of the 20 body regions tested, averaged across all participants. IF: index finger; MF: middle finger; LF: little finger; RF: ring finger. (DOC) [file pone.0045370.s002.doc]

| **Body Region** | **Mean Score (1-9)** |
| --- | --- |
| Thumb | 6.50 (2.09) |
| Lips | 6.32 (2.13) |
| IF | 6.26 (2.06) |
| Forehead | 6.00 (2.69) |
| Palm | 5.87 (2.54) |
| MF | 5.71 (2.22) |
| Breast | 5.71 (2.15) |
| Sole | 5.53 (2.41) |
| Cheek | 5.45 (2.23) |
| LF | 5.42 (2.52) |
| Shoulder | 5.39 (1.91) |
| Nose | 5.34 (2.29) |
| Belly | 5.32 (2.35) |
| Back | 5.24 (2.40) |
| RF | 5.13 (2.13) |
| Hallux | 4.97 (2.40) |
| Thigh | 4.92 (1.98) |
| Forearm | 4.49 (2.06) |
| Upper Arm | 4.08 (2.12) |
| Calf | 3.68 (2.07) |
